# Clomiphene citrate: A potential alternative for testosterone therapy in hypogonadal males

**DOI:** 10.1002/edm2.416

**Published:** 2023-03-30

**Authors:** M. Huijben, M. T. W. T. Lock, V. F. de Kemp, J. J. H. Beck, L. M. O. De Kort, H. M. K. van Breda

**Affiliations:** ^1^ Department of Urology University Medical Centre Utrecht Utrecht The Netherlands; ^2^ Department of Urology St Antonius Hospital Nieuwegein The Netherlands

**Keywords:** clomiphene citrate, male hypogonadism, selective oestrogen receptor modulator, testosterone deficiency syndrome

## Abstract

**Background:**

Hypogonadism is a worldwide problem among men causing sexual, physical and mental problems. Testosterone therapy is the first‐choice treatment for male hypogonadism, with several side effects, that is, subfertility. Clomiphene citrate (CC) is an alternative off‐label therapy for a certain group of hypogonadal males, especially for those with an active or future child wish. There is scarce literature in usage of CC for men with hypogonadism. The aim of this retrospective study was to evaluate the effectiveness and safety of CC for hypogonadal males.

**Methods:**

In this single‐centre study, men treated with CC for hypogonadism were evaluated retrospectively. Primary outcome was hormonal evaluation including total testosterone (TT), free testosterone (FT), luteinizing hormone (LH) and follicle stimulating hormone (FSH). Secondary outcomes were hypogonadal symptoms, metabolic and lipid parameters, haemoglobin (Hb), haematocrit (Ht), prostate specific antigen (PSA), side effects, the effect of a trial without medication and potential predictors for biochemical and clinical response.

**Results:**

In total, 153 hypogonadal men were treated with CC. Mean TT, FT, LH and FSH increased during treatment. TT increased from 9 to 16 nmol/L, with a biochemical increase in 89% of the patients. In patients who continued CC treatment, an increased level of TT persisted after 8 years of treatment. With CC treatment, 74% of the patients experienced hypogonadal symptom improvement. LH at the lower normal range before CC treatment was predictive for better TT response. During CC therapy, few side effects were reported and no clinical important changes in PSA, Hb and Ht were found.

**Conclusion:**

Clomiphene citrate is an effective therapy on short and long term, improving both clinical symptoms and biochemical markers of male hypogonadism with few side effects and good safety aspects.

## Graphical abstract

This retrospective study shows the good short and long‐term outcomes of clomiphene citrate on testosterone in hypogonadal males with good safety aspects.

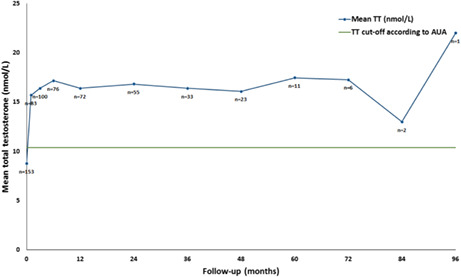

## 1 INTRODUCTION

Male hypogonadism is a biochemical and clinical testosterone deficiency syndrome with a prevalence of symptomatic hypogonadism ranging between 2.1% and 5.7% in males aged above 30 years of age.^1^, ^2^, ^3^, ^4^

Prevalence increases with age and certain comorbidities, such as cardiovascular disease, diabetes mellitus (DM), obesity and malignancies.^2^, ^5^ Decreased libido, lack of energy, mood changes, decreased muscle mass, and erectile dysfunction are common hypogonadal symptoms.^6^, ^7^ Testosterone therapy (TTh) is the treatment of first choice for male hypogonadism.^8^ However, exogenous testosterone leads to negative feedback on the hypothalamic–pituitary‐gonadal (HPG) axis, causing suppression of endogenous testosterone production and spermatogenesis.^9^ Other side effects are, changed lipid serum, polycythaemia and gynecomastia.^9^, ^10^, ^11^

Clomiphene citrate (CC) is an alternative off‐label pharmacological treatment for a certain group of males with hypogonadism. CC is a selective oestrogen receptor modulator, occupying oestrogen receptors in the hypothalamus and pituitary gland, leading to increased secretion of luteinizing hormone (LH) and follicle stimulating hormone (FSH) and so, stimulating testicular endogenous testosterone production and preserving spermatogenesis.^12^, ^13^

Since the 1960s, CC has been often used for ovulation induction in females. For males, the United States Food and Drug Administration (FDA) did not approve the medicine with reason of unclear clinical effect because of the lack of well‐controlled and well‐powered controlled trials.^14^, ^15^, ^16^, ^17^ However, CC is described over more than 30 years off‐label to hypogonadal men, especially those with an active or future child wish, or hypogonadal males who do not want to use TTh.^18^ Compared to TTh, CC is easy in usage and comes with little costs. For example, in our country, costs of CC are about 12 times lower than regular TTh.^19^

To support the outcomes in the scarce literature on the efficacy of CC therapy, the purpose of this retrospective study was to evaluate the effectiveness and safety of CC therapy for male hypogonadism.

## 2 METHODS

### 2.1 Study design

A retrospective, single‐centre study was conducted in the University Medical Center (UMC) Utrecht. Hypogonadal men, who wanted to preserve their endogenous testicular function or men who had other reasons for not willing to use TTh and therefore treated with CC within the time frame from January 2012 to January 2021 were included. Ethical approval for this retrospective study was obtained from the local Research Ethics Committee, UMC Utrecht, the Netherlands (WAG/mb/20/500309). All patients were informed that CC is used as an off‐label therapy.

### 2.2 Patient population

Men aged above 18 years with low (<12.1 nmol/L, defined by the EAU guidelines)^3^ or relatively low TT in combination with clear hypogonadal symptoms, wherefore treatment with CC, were included. Relatively low testosterone was defined as testosterone <15 nmol/L in combination with, (1) low free testosterone <243 pmol/L or (2) young adult males with a history of orchiectomy and clear complaints of hypogonadism. No upper cut‐off values for FSH nor LH levels were used for inclusion. Patients with LH levels >10 IU/L were classified as hypergonadotropic hypogonadal males.^20^

Hypogonadal symptoms were scored by the experienced treating physicians, no validated questionnaires were used. Patients were excluded in case: hypogonadotropic hypogonadism, no assessment of TT/FT was done before or during treatment, CC was used in combination with TTh, human chorionic gonadotrophin (hCG) or aromatase inhibitors (AI) or if these therapies were used within 12 months of CC initiation.

With the clinical knowledge in 2012 based on expert opinion at that time, men received CC, with a starting dosage of 25 mg every other day, or 25 mg a day in case of bodyweight above 100 kg. Dosage was raised to 50 mg daily when there was little biochemical response. Data of duration of CC therapy, patient characteristics (age, body mass index (BMI), obesity (BMI >25 kg/m))^2^, testicular volume, medical history (HIV, testicular surgery, DM, cardiovascular disease, diagnosis of Klinefelter syndrome, malignancies, polycythaemia) and additional medication for erectile dysfunction during treatment were obtained manually from patient files.

### 2.3 Primary outcomes

Study primary outcome was hormonal assessment at baseline and at 1, 3, 6 and 12 months during CC therapy and annually thereafter measured with immunoassay (Atellica®, Siemens, Erlangen, Germany) the immunoassay was replaced in 2014, however, hormonal values were comparable using both immunoassay tools:

1. Total testosterone (TT) from early morning blood draws (<11 am) (laboratory standard (lab. st.): 7.0–31 nmol/L)
2. Free testosterone (FT) from early morning blood draws (<11 am) (lab. st.: 230–600 pmol/L)
3. LH (lab. st.: 1.0–9.0 IU/L)
4. FSH (lab. st.: 1.0–19.0 IU/L)
5. Estradiol (lab. st.: 70–200 pmol/L)
6. Sex‐hormone binding globulin (SHBG) (lab. st.: 10–40 nmol/L)
7. Albumin (lab. st.: 35.0–50.0 g/L)
8. Prolactin (lab. st.: 0.10–0.65 IU/L)

### 2.4 Secondary outcomes

1. Presence of hypogonadal symptoms measured at baseline. Subjective symptom improvement was evaluated during follow‐up (FU).
2. BMI, HbA1c, serum total cholesterol, triglycerides, low‐density lipoprotein (LDL)‐, high‐density lipoprotein (HDL)‐ and non‐HDL cholesterol, before and during treatment.
3. Self‐reported side effects were noted during treatment.
4. Haematocrit (Ht), haemoglobin (Hb), thrombocytes, alanine aminotransferase (ALAT), aspartate aminotransaminase (ASAT), alkalic phosphatases (AF), gamma‐glutamyl transferase (gGT) and total prostate‐specific antigen (PSA) before and during treatment.
5. Data on the presence of a trial without CC therapy, the reason and duration of trial without medication (TWM), TT, FT and symptom changes during TWM, and whether patients restart CC therapy after TWM, were collected.
6. The classification of biochemical responder, non‐responders or a reversed effect on TT. Patients were classified as biochemical responder if there was a significant increase in TT and/or FT. Non‐responders were classified if there was no change in TT and/or FT. A reversed effect was defined as a significant decrease in TT and/or FT.

### 2.5 Data analysis

Data were recorded and tabulated with Microsoft Excel® software. Values were presented as number (%), mean (standard deviation [SD]) or median (25th–75th percentile interquartile range [IQR]) if data were not normally distributed. Normality of continuous variables was tested with the Shapiro–Wilk‐test, histograms and normality‐quantile plots. Paired sample t‐test was used to compare before‐ and during treatment hormonal levels, safety aspects, serum lipids, BMI. The Wilcoxon signed ranked test was used if the data did not meet the assumptions for normality. Assumptions for correlation analysis were normality, homoscedasticity and linearity. The Pearson test was used for bivariate correlation, if data did not meet the assumption for normality the Spearman test was used. A *p*‐value ≤.05 was considered statistically significant. Statistical analysis was performed by using SPSS (IBM SPSS Statistics, Version 25.0. Armonk, NY: IBM Corp.).

## 3 RESULTS

### 3.1 Study population

In total, 173 male patients treated with CC were screened for inclusion. Twenty patients were excluded with reason; four used CC combined with TTh or hCG, six had no wash‐out period longer than 12 months between TTh and before start of CC therapy, two had no before treatment TT and/or FT measurement and eight were lost to FU before any hormonal evaluation. Finally, 153 patients were included for analysis. Data on patient characteristics, comorbidities and dosage at start of treatment, are provided in Table 1. Forty‐one patients had an orchiectomy in the past, 22/41 patients (54%) underwent adjuvant chemotherapy, radiation therapy or a combination of both. Median duration of CC therapy was 10 months (range, 1–96). During the first year of CC, 21 patients (14%) were lost to FU and 50 patients (33%) stopped treatment. At the end‐point of this study (January 2021), 43 patients (28%) were still in FU, 26 patients (17%) were lost to FU, 71 patients (46%) stopped CC therapy and 14 patients (9%) stopped CC and switched to TTh. There was no correlation between discontinuation in the first year and pre‐treatment TT levels, age or hypogonadism aetiology. Dosage was increased (*n* = 22, 14%) in case of minimal TT response or decreased in case of side effects within the first year (*n* = 10, 7%). In 145 patients (95%) pre‐treatment TT levels were <12.1 nmol/L. Two patients (1%) had TT level <15 nmol/L, but FT levels <243 pmol/L. Six patients (4%) had TT levels <15 nmol/L and FT levels <280 pmol/L. All of these eight patients with TT levels >12 nmol/L and < 15 nmol/L had clear hypogonadal symptoms.

**TABLE 1:** Patient characteristics at baseline.

Characteristics	Total 153 patients	
Age, mean ± SD (range), years	48.6 ± 12.8 (22–80)	(*n* = 153)
Overweight (BMI >25 kg/m^2^), *n* (%)	75 (67)	(*n* = 112)
Testis volume		(*n* = 75)
Left testis, median (IQR)	18.0 (15.0–20.0)	
Right testis, median (IQR)	18.0 (15.0–18.0)	
Medical history		(*n* = 153)
Klinefelter, *n* (%)	1 (1)	
Orchiectomy, *n* (%)	41 (27)	
Orchidopexy, *n* (%)	5 (3)	
Vasectomy, *n* (%)	17 (11)	
Vasovasostomy, *n* (%)	3 (2)	
Diabetes mellitus, *n* (%)	15 (10)	
Testicular tumour, *n* (%)	38 (23)	
Other malignancies*, *n* (%)	16 (10)	
Pituitary adenoma, *n* (%)	2 (1)	
HIV, *n* (%)	3 (2)	
Myocardial or brain infarction, *n* (%)	16 (10)	
Hypertension, *n* (%)	19 (12)	
Thrombosis or lung embolus, *n* (%)	5 (3)	
Polycythaemia, *n* (%)	2 (1)	
Dosage of CC therapy at start		(*n* = 153)
25 mg/2 day	132 (86)	
25 mg/day	9 (6)	
50 mg/2 day	6 (4)	
50 mg/day	3 (2)	
25 mg/3 day	1 (1)	
Switching between 25 or 50 mg/day	1 (1)	

Abbreviations: BMI, body mass index; IQR, interquartile range; *n*, number of patients; SD, standard deviation.

* Prostate carcinoma, renal cell carcinoma, lymphoma, Kaposi sarcoma, colon carcinoma, rectum carcinoma.

### 3.2 Hormonal evaluation

**FIGURE 1:**
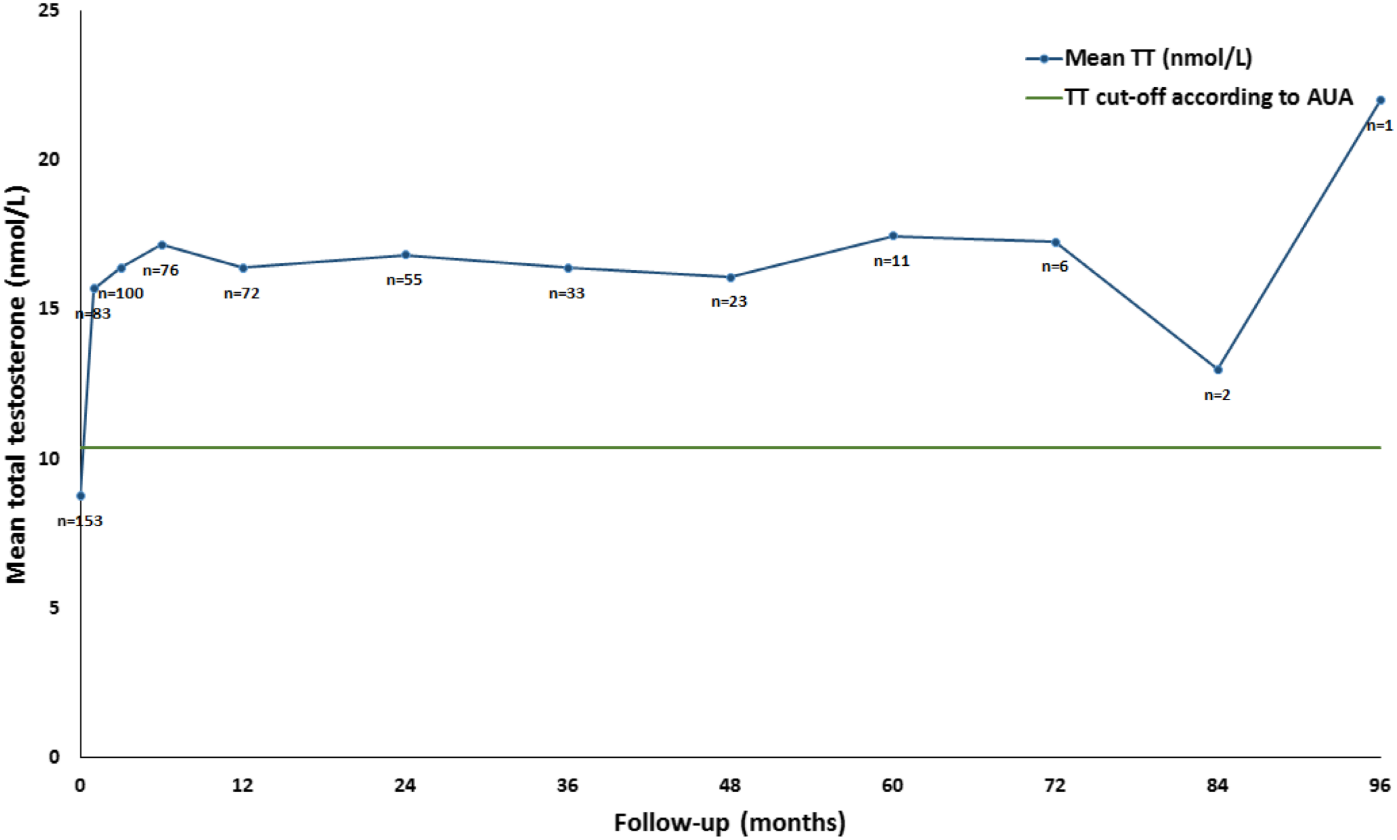
Long‐term effect of clomiphene citrate (CC) therapy on total testosterone (TT).

Data of hormonal levels are provided in Table 2. The first measurement during treatment was at a median of 1.5 months (range, 1–12). Levels of TT, FT, LH, FSH and SHBG increased, and albumin decreased at first measurement during treatment and annually thereafter till 8 years of FU (*p* ≤ .05) (Figures 1. and 2). All eight patients with TT levels >12.1 nmol/L and <15 nmol/L showed increased levels of TT and FT. At first measurement during treatment, median TT increased from 9 to 16 nmol/L, with a biochemical increase in 136 patients (89%) during CC treatment (Table 3). Fifteen patients (10%) were biochemical non‐responders, these patients stopped treatment between 1 and 6 months after starting CC therapy. In this non‐responder group, mean LH before treatment was 13.7 IU/L (range, 4.6–29), compared with a mean of 4.7 IU/L (range, 0.6–25) in the responder group.

**TABLE 2:** Hormonal evaluation before and during CC treatment. Data are tabulated as mean ± SD or median (IQR).

	Before CC treatment	First measurement of CC treatment	1 year with CC treatment	2 year with CC treatment	3 year with CC treatment	4 year with CC treatment	5 year with CC treatment	6 year with CC treatment	7 year with CC treatment	8 year with CC treatment
TT, nmol/L	8.8 ± 3.1 (*n* = 153)	16.0 (12–20)* (*n* = 153)	16.0 (12–19)* (*n* = 72)	16.8 ± 4.4* (*n* = 55)	16.4 ± 5.7* (*n* = 33)	16.1 ± 5.0* (*n* = 23)	17.5 ± 5.0* (*n* = 11)	17.3 ± 5.7* (*n* = 6)	13.0 (10–16) (*n* = 2)	22.0 (*n* = 1)
FT, pmol/L	178.6 ± 55.7 (*n* = 149)	340.0 (240–430)* (*n* = 148)	310.0 (270–410)* (*n* = 71)	332.3 ± 99.5* (*n* = 52)	331.3 ± 119.3* (*n* = 31)	334.3 ± 113.4* (*n* = 21)	330.0 (290–420)* (*n* = 11)	326.7 ± 166.5 (*n* = 6)	290.0 (200–380) (*n* = 2)	n.a.
LH, IU/L	4.0 (2.4–6.8) (*n* = 138)	7.7 (5.0–13.8)* (*n* = 144)	7.0 (3.9–11.3)* (*n* = 58)	7.6 (5.3–11.3)* (*n* = 38)	9.8 ± 4.9* (*n* = 22)	8.9 ± 5.0* (*n* = 16)	9.2 ± 4.5* (*n* = 10)	6.8 ± 2.7* (*n* = 5)	7.1 (4.1–10.0) (*n* = 2)	5.1 (*n* = 1)
FSH, IU/L	7.3 (4.3–15.8) (*n* = 136)	11.0 (7.2–26.0)* (*n* = 143)	10.4 (6.7–19.8)* (*n* = 56)	12.0 (6.9–16.3)* (*n* = 38)	12.0 (7.2–19.0)* (*n* = 23)	9.5 (5.6–22.0)* (*n* = 15)	15.5 (7.6–23.3)* (*n* = 10)	12.2 ± 5.1* (*n* = 4)	15.8 (7.5–24) (*n* = 2)	11.0 (*n* = 1)
Estradiol, pmol/L	76.7 ± 35.6 (*n* = 13)	122.5 ± 51.4 (*n* = 17)	119.5 (84.0–135.5) (*n* = 4)	181.0 (120.0–181.0) (*n* = 3)	120.5 (86.5–138.0) (*n* = 4)	78.5 (21.0–136.0) (*n* = 2)	n.a.	n.a.	n.a.	n.a.
SHBG, nmol/L	30.5 (22.0–42.3) (*n* = 150)	36.0 (24.0–49.0)* (*n* = 150)	38.5 (27.0–54.0)* (*n* = 72)	43.3 ± 16.1* (*n* = 51)	40.5 ± 16.2* (*n* = 32)	40.0 (28.0–48.5)* (*n* = 21)	38.7 ± 9.1* (*n* = 11)	53.0 ± 30.1* (*n* = 6)	36.0 (30.0–42.0) (*n* = 2)	20.0 (*n* = 1)
Albumin g/L	44.1 (41.7–45.7) (*n* = 148)	43.1 (41.1–44.5)* (*n* = 146)	42.8 ± 2.5* (*n* = 73)	42.4 (40.1–44.2)* (*n* = 52)	41.7 ± 2.4* (*n* = 33)	41.3 ± 2.3* (*n* = 21)	40.8 ± 1.7 (*n* = 11)	40.5 ± 2.8 (*n* = 6)	39.5 (38.6–40.4) (*n* = 2)	n.a.
Prolactin, IU/L	0.17 (0.13–0.25) (*n* = 45)	0.17 (0.12–0.22) (*n* = 39)	n.a.	n.a.	n.a.	n.a.	n.a.	n.a.	n.a.	n.a.

Abbreviations: CC, clomiphene citrate; FSH, follicle stimulating hormone; FT, free testosterone; LH, luteinizing hormone; *n*, number of patients; n.a, not available; SD, standard deviation; SHBG, sex hormone binding globulin; TT, total testosterone.

* *p*  ≤ .05.

**FIGURE 2:**
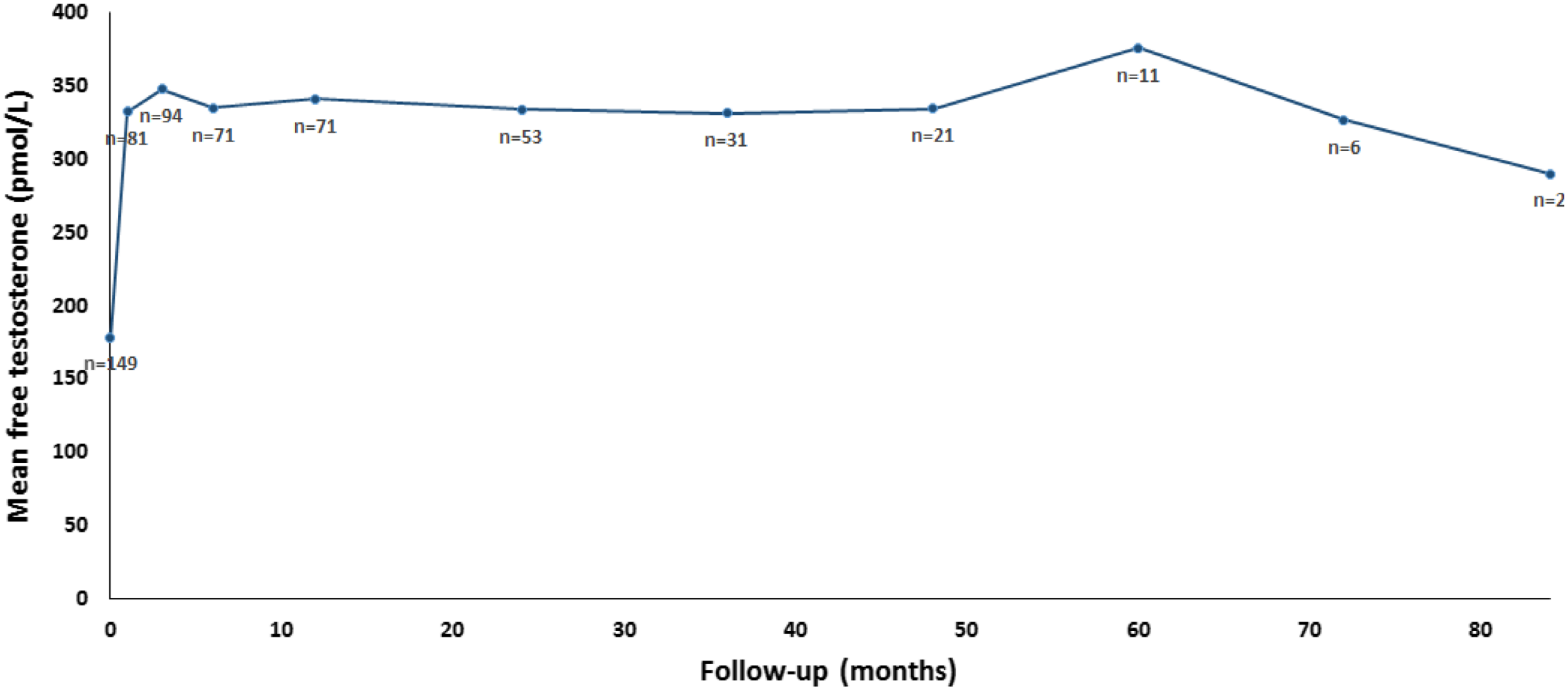
Long‐term effect of clomiphene citrate (CC) therapy on free testosterone (FT).

**TABLE 3:** Biochemical and clinical response of CC therapy and hypogonadal symptom improvement.

Total population of biochemical response (*n* = 153)	Total population of clinical response (*n* = 151)	
	Symptom improvement (74%)	no symptom improvement (26%)
Biochemical responders (*n* = 136%, 89%)	81%	19%
Biochemical non‐responders (*n* = 15%, 10%)	27%	73%
Reversed TT response (*n* = 2%, 1%)	0%	100%

Hypogonadal self‐reported symptoms	Before CC treatment presence of symptoms (without *n* = 151)	During treatment improvement of symptoms
Decreased libido	86 (57)	43 (50)
Erectile dysfunction	84 (56)	44 (52)
Fatigue	79 (52)	57 (72)
Depressive thoughts	11 (7)	n.a
Decreased energy	10 (6)	n.a
Weak muscle strength	6 (4)	n.a
Gynecomastia	5 (3)	n.a
Mood changes	4 (3)	n.a
Agitation	3 (2)	n.a

Abbreviations: CC, clomiphene citrate; *n*, number of patients; n.a, not available.

Sixteen patients (10%) were classified before treatment as hypergonadotropic hypogonadism. In this hypergonadotropic hypogonadism group 6/16 were biochemical responders (38%), of which the patient with Klinefelter. The Klinefelter patient showed an increase in TT of 6 nmol/L tot 14 nmol/L and FT from 140 pmol/L to 280 pmol/L during CC treatment. In the orchiectomy patient group, 11/41 patients were hypergonadotropic, and 9/11 patients hypergonadotropic hypogonadal orchiectomy patients were biochemical non‐responders. Two hypergonadotropic patients (1%) had a reversed response of TT after start of CC therapy (see section 4.8).

### 3.3 Hypogonadal symptoms

Table 3 describes hypogonadal symptoms before and during treatment. Symptoms at baseline were reported in 151 patients, in two patients there was, besides a low testosterone, no clarity in the presence of symptoms before treatment. During treatment, 74% of the patients had subjective symptom improvement, with a median duration till improvement of 3 months (range, 1–24). Symptom improvement was noticed in 81% of the biochemical responder patients, and in 27% of the biochemical non‐responder patients. Six of eight patients with TT levels >12 nmol/L but <15 nmol/L showed improvement of hypogonadal symptoms during CC therapy. The patient with Klinefelter described an improvement of fatigue during CC treatment. In the orchiectomy patient group with hypergonadotropic hypogonadism (*n* = 11) only the two biochemical responders described symptom improvement.

### 3.4 Metabolic and lipid parameters

Total cholesterol, LDL‐, HDL‐ and non‐HDL cholesterol decreased during treatment (*p* < .05). However, it is unclear if all the values were based on fasting blood tests. BMI and triglycerides did not change before and during treatment. Data are available in of the Appendix S1–S2.

### 3.5 Safety aspects and side effects

There were no changes in Ht, Hb, thrombocytes, liver enzymes and PSA (in Appendix S2). Two patients had marginally elevated Ht during treatment (0.51 L/L), without availability of baseline Ht levels. In one patient, CC therapy was stopped after detecting this elevated Ht (0.51). In the other patient with Ht 0.50, CC therapy was continued and Ht values were monitored. One patient, who had a history of lower extremity thrombosis, developed a lung embolus in the fourth year of FU. In 16 patients (10%) side effect were present, hot flushes (*n* = 5), agitation (*n* = 4), visual changes (*n* = 2), nipple tenderness (*n* = 2), mood change (*n* = 1), weight gain (*n* = 1), blurred vision (*n* = 1), headache (*n* = 1), dizziness (*n* = 1).

### 3.6 Trial without medication

Fifty‐two patients had a trial without CC therapy (34%), with a median duration of 3 months (range, 1–12). Reasons for TWM were; physician‐initiated testing of CC effect (*n* = 44, 85%), presence of side effects (hot flushes, agitation, mood disorder) (*n* = 4, 8%), run out of medication (*n* = 3, 6%), or extremely elevated estradiol (*n* = 1, 2%). During TWM, TT decreased significant in 48 of these 52 patients (94%), with a mean TT during CC treatment of 19.1 nmol/L(5.6) and during TWM of 10.8 nmol/L (3.1) (*p* = .000). Of these patients in whom TT decreased during TWM, 22 patients were obese and 16 patients had an orchiectomy in the past of which two were hyperchonadotropic. The 4/52 patients in whom TT did not decreased, three of them had an orchiectomy in the past, two patients had TT levels between >12 nmol/L and 15 nmol/L before start treatment and LH levels before start of treatment were in all patients not available. Mean FT during CC therapy was 373.2 pmol/L (126.6) and during TWM 203.3 pmol/L (63.5) (*p* = .000). Clinically, 20/52 patients noticed a worsening of symptoms during TWM (38%), fatigue (*n* = 12), libido (*n* = 8), depressive thoughts (*n* = 3), erectile dysfunction (*n* = 2), agitation (*n* = 2). Eighteen /52 patients (35%) restarted CC therapy after the TWM, three of them were the patients with side effects. In 44% of the patients without noticing a difference in symptoms during TWM, TT levels were >12 nmol/L during TWM. Eight/35 patients (25%) who did not restart CC after TWM had TT levels before initiating CC treatment of >12 nmol/L. There was no correlation between discontinuation CC therapy and pre‐treatment TT levels, age or hypogonadism aetiology. Only 14/35 (32%) of the physician‐initiated TWM patients continued CC therapy after TWM.

### 3.7 Correlations

A correlation was found between symptom improvement during treatment and a higher increase in TT at first measurement during treatment (*p* = .000, *r* = .34). A second correlation was found between a lower range of normal LH and FSH before treatment and a higher increase in TT at first measurement during treatment (*p* = .000, *r* = −.37 and *p* = .000, *r* = −.40, respectively). A third correlation was found between a low‐normal LH before treatment and improvement of symptoms with CC therapy (*p* = .02, *r* = −.21). Further, a correlation was found between a lower increase in TT at first measurement and an orchiectomy in the medical history (*p* = 0.009, *r* = −0.21). No correlations were found between first measurement during TT treatment and age, BMI, testis volume, and different subgroups of patients with a presence/history of overweight, cardiovascular disease, chemo‐ and/or radiation therapy, DM, Klinefelter, other malignancies.

### 3.8 Reversed effect of CC

Two patients had a reversed biochemical effect within the first 3 months of CC treatment. Patients were 30 and 40 years old, both with a history of orchiectomy for seminoma. The patients used CC therapy 25 mg every other day. TT levels before treatment were 3.7 and 6.1 nmol/L, respectively and during CC treatment 0.5 and 1.3 nmol/L, respectively. LH levels before treatment were 26 and 27 IU/L, respectively and during CC treatment 8.6 and 9.0 IU/L respectively. Before treatment, both patients had fatigue and decreased libido, one patient had erectile dysfunction. Neither improvement nor worsening of symptoms occurred during CC treatment. Treatment was stopped after a second blood test confirmed this reversed effect. After stopping CC therapy, both patients had restoration to before treatment levels of testosterone.

## 4 DISCUSSION

This study is supporting evidence for the long‐term efficacy of CC in hypogonadal males with increases in total testosterone, free testosterone and gonadotrophins and a total biochemical increase in TT in 89% of the patients. Therewithal, in 74% of patients, a clinical effect was observed as described as an improvement of hypogonadal symptoms with CC therapy. In total 81% of the patients had both a biochemical and clinical response. This study is, to our knowledge, the first large retrospective cohort study on both clinical and biochemical effect of CC in hypogonadal males with long follow‐up and identification of potential predictors for success.

Levels of TT, FT, LH, FSH and SHBG increased after start treatment. Increased testosterone levels lasted with ongoing treatment until 8 years of FU. This improvement of hormone levels has been described in previous studies, showing TT at baseline ranging between 7 and 11 nmol/L and at first measurement during treatment ranging between 16 and 24 nmol/L (*n* = 34–400).^21^, ^22^, ^23^, ^24^ The results of testosterone and gonadotrophins show that CC is effective in increasing endogenous testosterone secretion by stimulating the HPG axis in hypogonadal men. Only 10% of our study population was biochemical non‐responder, all these patients stopped CC therapy during the first 6 months. In the study, mean LH before treatment was 4.7 IU/L in the responder group and 13.7 IU/L in the non‐responder group. Of the 16 patients with hypergonadotropic patients, six were biochemical responders. Of which, the patient with Klinefelter who showed a biochemical and symptomatic improvement.

For further research and practice it is important to be critical who to include or to prescribe CC therapy. However with these results, it could be considered to try CC therapy also in hypergonadotropic patients. Although, it could be recommended then to do an early testosterone measurement, to evaluate the biochemical response and for early detection of a potential reversed effect. Furthermore, it is debatable if patients with TT levels >12 nmol/L and < 15 nmol/L should be included. However, in all these eight patients there was a biochemical improvement and in six of them (75%) there was clinical improvement (increased libido). Considering of inclusion of these patients is supported by the study of Zitzmann (2006), showing that testosterone deficiency symptoms may also be seen with TT levels as high as 15 nmol/L.^25^

It is unknown whether CC is effective on the long term. In our study, there was a sustained biochemical effect of CC, with the longest long‐term FU of 8 years. Three earlier studies (*n* = 29–120) indicated that after 24–52 months of CC use, hormone levels were stable.^21^, ^26^, ^27^ It is worth focusing on the 52 patients who underwent a TWM to gain further insight in this long‐term effect. In the current study, there was a decrease in TT levels <15 nmol/L after stopping CC in 94% of the patients and in 65% of the patients <12.1 nmol/L. This effect was also seen by Marconi et al. (2016) (*n* = 27) demonstrating during trial without CC therapy that 78% of the patients had decreased TT levels of <10 nmol/ after 3 months, and all patients had decreased TT levels of <10 nmol/L after 6 months.^28^ Remarkably, only 38% of patients in the current study reported worsening of hypogonadal symptoms while on TWM and only 32% of the TWM patients continued CC therapy after TWM. Furthermore, in the current study, 33% of patients ceased CC treatment in the first year and almost half of them by the end of the study, with no correlation between discontinuation in the first year and pre‐treatment TT levels, age or hypogonadism aetiology. Despite improvement of TT levels, most mentioned reason for stopping was that patients did not experience enough effects of CC treatment. A possible explanation for not continuing CC therapy after TWM is that the symptoms were not impeding them as much as they did before treatment start and during the first time of treatment. Either, the symptoms were mild or patients life or relational situation changed and the hypogonadal symptoms hindered them less than before and not enough to take long‐term medication. Further studies should consider stricter inclusion criteria such as low testosterone levels (<12.1 nmol/L) and use validated symptom questionnaires to objective symptom severity.

Furthermore, this retrospective study is the first study showing that a better early response of TT is correlated with a better clinical response. The study also shows that low‐normal LH before treatment predicted symptom improvement and a higher increase in TT during CC treatment. This potential predictor was also seen in the study of Mazzola et al. (2014).^29^ Without any confirmed mechanism, it is hypothesized that low normal range LH levels demonstrate a better potential for stimulating Leydig cells by CC. Further predictors were demonstrated by Guay et al. (2003) (*n* = 178) who showed that younger age (<55 years) and absence of diabetes is a predictor for better TT response.^30^ This was not seen in our study. In another study specific enzymes like CYP450 D26 were suggested as predictors for TT response.^31^ In our study those enzymes were not measured. Also in earlier studies on CC, CYP450 D26 was not measured.

Side effects were reported in 10% of our population and included agitation, hot flushes, nipple tenderness, mood changes, weight gain, visual changes, headache and dizziness. These side effects were also reported in the literature, with a prevalence between 4% and 11%.^21^, ^23^, ^31^, ^32^ Most patients with side effects continued CC after the TWM, suggesting that CC's benefits outweighed side effects. Because of the off‐label usage of CC, there are no existing guidelines for monitoring adverse events. Therefore, recommendations are based on experience with exogenous testosterone therapy. Literature shows that TTh can lead to an increase in PSA, Hb and Ht.^10^, ^33^ In our study we found no increase in PSA and Hb, only two patients showed marginal elevated Ht (0.50 and 0.51) during treatment. Baseline Ht levels were not available in these patients, so it is unclear if this elevated Ht was pre‐existent. This was supported by Chandrapal et al. (2016) who demonstrated no increase in PSA, Hb and Ht during CC therapy (*n* = 77).^34^ This was further supported by Krzastek et al. (2019) (*n* = 76), who found only one patient with increased Ht (0.51) during CC therapy.^21^ Wheeler et al. (2017) demonstrated a difference in prevalence of increased Ht with CC and TTh of 2% and 11%, respectively (*n* = 363).^35^ A hypothesis is, that rapid peak increases in TT and overdosing of TT, especially in TTh injections, can lead to treatment‐induced increase in Hb and Ht.^36^ Thus, this does not apply for CC therapy, with slow increase in endogenous TT. However, CC can have an oestrogen agonistic activity, leading to activation of clotting factors, leading to an increased coaguliability, with increased risk of thromboembolic events.^37^ Although, with this theoretical risk in mind, Kavoussi et al. (2019) showed that the risk of deep venous thrombosis was not increased with CC compared to the general population (*n* = 1180)^38^ It could be considered that patients should be counselled that there are some described cases of thromboembolic events without the knowledge of this was caused by CC. Furthermore, it could be considered that an elevated haematocrit before treatment or thromboembolic events in the past are contraindications. In our study an increase in estradiol was only seen in (1/13) patient where estradiol was measured before and during treatment, in contrast with previous studies.^21^, ^22^, ^29^, ^31^ A possible explanation could be that estradiol was measured in only a small number of our study population (*n* = 13).

A reversed effect of CC on testosterone levels was found in two young hypergonadotropic patients (<1%). Although this phenomenon has been described before in the literature, the physiology of this paradoxical effect of CC is unclear.^21^, ^39^, ^40^

There are several limitations to address for this study. First, it has a retrospective character, with data obtained manually from patient files. There were no strict inclusion criteria with predefined baseline and outcome measurements. No validated instrument for reporting hypogonadal symptoms could be used because there does not exist a Dutch validated hypogonadism questionnaire, but self‐reported hypogonadal symptoms and subjective symptom improvement was evaluated during follow‐up. Second, because this was not a placebo controlled trial, it is difficult to conclude if clinical improvement is the result of CC therapy. Third, in 130 patients, the presence of side effects was not reported, and side effects were not systematically classified. This may have caused an underestimation of the actual prevalence of side effects. Fourth, dosage at start of therapy was chosen based on weight, based on expert opinion in 2012. For future research and for clinical practice it is recommended with this gap in knowledge to start with the minimal dosage not depending on weight and to titrate dosage up if there is no sufficient effect. As last to mention, the included patients had different origins of hypogonadism. This is causing heterogeneity and does not imply that the effect of the study do count for all different subgroups. Nevertheless, with the encouraging outcomes of this study, we believe it is very valuable to execute a prospective study, especially for young hypogonadal males with an active or potential future child wish who are not eligible for TTh, with clear inclusion criteria, adequate measurement of hormonal levels, symptomatology with the usage of a validated questionnaire and registration of side effects.

## 5 CONCLUSION

Clomiphene citrate therapy for hypogonadal males shows promising effect on both clinical symptoms and the biochemical testosterone insufficiency with few reported side effects and good safety aspects compared with TTh. Therefore, it is worth to be considered especially in males presenting with symptoms of hypogonadism and low testosterone who wish to preserve their testicular function and are not eligible for TTh. Low to normal LH at baseline seems to predict biochemical and clinical effectiveness. Further research with clear inclusion criteria, adequate measurement of hormonal levels and registration of symptoms and side effects is recommended.

## AUTHOR CONTRIBUTIONS

M.H., T.L., V.d.K., J.B., L.d.K., H.v.B., contributed to the design, to the analysis of the results and to the writing of the manuscript.

## FUNDING STATEMENT

The authors did not receive funding for this study.

## CONFLICT OF INTEREST STATEMENT

There is no conflict of interest.

## Supporting information


Appendix S1–S2.

## Data Availability

Data on data collection and analysis are available on request.

## References

[1] Nieschlag E , Behre HM , Nieschlag S . In: Nieschlag E , Behre HM , Nieschlag S , eds. Andrology. Berlin, Heidelberg, Springer Berlin Heidelberg; 2010.

[2] Araujo AB , Esche GR , Kupelian V , et al. Prevalence of symptomatic androgen deficiency in men. J Clin Endocrinol Metab. 2007;92:4241‐4247. 17698901 10.1210/jc.2007-1245

[3] Dohle G , Arver STE , Bettocchi C , Jones TH , Kliesch S . EAU Guidelines on Male Hypogonadism, Edn. presented at the EAU Annual Congress Barcelona 2019. ISBN 978‐94‐92671‐04‐2. EAU Guidelines Office, Arnhem, The Netherlands. http://uroweb.org/guideline/male‐hypogonadism/

[4] Khera M , Adaikan G , Buvat J , et al. Diagnosis and treatment of testosterone deficiency: recommendations from the fourth international consultation for sexual medicine (ICSM 2015). J Sex Med. 2016;13:1787‐1804. 27914560 10.1016/j.jsxm.2016.10.009

[5] Zarotsky V , Huang MY , Carman W , et al. Systematic literature review of the risk factors, comorbidities, and consequences of hypogonadism in men. Andrology. 2014;2:819‐834. 25269643 10.1111/andr.274

[6] Hall SA , Esche GR , Araujo AB , et al. Correlates of low testosterone and symptomatic androgen deficiency in a population‐based sample. J Clin Endocrinol Metab. 2008;93:3870‐3877. 18664536 10.1210/jc.2008-0021 PMC2579652

[7] Wu FCW , Tajar A , Beynon JM , et al. Identification of late‐onset hypogonadism in middle‐aged and elderly men. N Engl J Med. 2010;363:123‐135. 20554979 10.1056/NEJMoa0911101

[8] Traish AM . Benefits and health implications of testosterone therapy in men with testosterone deficiency. Sex Med Rev. 2018;6:86‐105. 29128268 10.1016/j.sxmr.2017.10.001

[9] Pastuszak AW , Gomez LP , Scovell JM , Khera M , Lamb DJ , Lipshultz LI . Comparison of the effects of testosterone gels, injections, and pellets on serum hormones, erythrocytosis, lipids, and prostate‐specific antigen. Sex Med. 2015;3:165‐173. 26468380 10.1002/sm2.76 PMC4599554

[10] Saad F , Aversa A , Isidori AM , Zafalon L , Zitzmann M , Gooren L . Onset of effects of testosterone treatment and time span until maximum effects are achieved. Eur J Endocrinol. 2011;165:675‐685. 21753068 10.1530/EJE-11-0221 PMC3188848

[11] Gagnon DR , Zhang TJ , Brand FN , Kannel WB . Hematocrit and the risk of cardiovascular disease‐the Framingham study: a 34‐year follow‐up. Am Heart J. 1994;127:674‐682. 8122618 10.1016/0002-8703(94)90679-3

[12] Surampudi P , Swerdloff RS , Wang C . An update on male hypogonadism therapy. Expert Opin Pharmacother. 2014;15:1247‐1264. 24758365 10.1517/14656566.2014.913022 PMC4168024

[13] Huijben M , Huijsmans RLN , Lock MTWT , de Kemp VF , de Kort LMO , van Breda JHMK . Clomiphene citrate for male infertility: A systematic review and meta‐analysis. Andrology Published online January 21. 2023:1‐10. doi:10.1111/andr.13388 36680549

[14] Wiehle R , Cunningham GR , Pitteloud N , et al. Testosterone restoration using enclomiphene citrate in men with secondary hypogonadism: a pharmacodynamic and pharmacokinetic study. BJU Int. 2013;112:1188‐1200. 23875626 10.1111/bju.12363 PMC4155868

[15] Rodriguez KM , Pastuszak AW , Lipshultz LI . Enclomiphene citrate for the treatment of secondary male hypogonadism. Expert Opin Pharmacother. 2016;17:1561‐1567. 27337642 10.1080/14656566.2016.1204294 PMC5009465

[16] Bone, Reproductive and Urologic Drugs Advisory Committee . ADVISORY COMMITTEE INDUSTRY BRIEFING DOCUMENT. 2016 https://fda.report/media/101593/Industry‐Briefing‐Information‐for‐the‐December‐6‐‐2016‐Meeting‐of‐the‐Bone‐‐Reproductive‐and‐Urologic‐Drugs‐Advisory‐Committee.pdf Accessed June 12, 2021.

[17] Huijben M , Lock MTW , de Kemp VF , de Kort LM , van Breda HMK . Clomiphene citrate for men with hypogonadism: a systematic review and meta‐analysis. Andrology. 2022;10(3):451‐469. 34933414 10.1111/andr.13146

[18] Kaminetsky J , Hemani ML . Clomiphene citrate and enclomiphene for the treatment of hypogonadal androgen deficiency. Expert Opin Investig Drugs. 2009;18:1947‐1955. 10.1517/13543780903405608 19938905

[19] Medicijnkosten.nl. https://www.medicijnkosten.nl/ Accessed May 12, 2021.

[20] Bjerner J , Biernat D , Fosså SD , Bjøro T . Reference intervals for serum testosterone, SHBG, LH and FSH in males from the NORIP project. Scand J Clin Lab Invest. 2009;69(8):873‐879. 19929279 10.3109/00365510903380886

[21] Krzastek SC , Sharma D , Abdullah N , et al. Long‐term safety and efficacy of clomiphene citrate for the treatment of hypogonadism. J Urol. 2019;202:1029‐1035. 31216250 10.1097/JU.0000000000000396

[22] Soares AH , Horie NC , Chiang LAP , et al. Effects of clomiphene citrate on male obesity‐associated hypogonadism: a randomized, double‐blind, placebo‐controlled study. Int J Obes (Lond). 2018;42:953‐963. 29777228 10.1038/s41366-018-0105-2

[23] Da Ros CT , Averbeck MA . Twenty‐five milligrams of clomiphene citrate presents positive effect on treatment of male testosterone deficiency ‐ a prospective study. Int Braz J Urol. 2012;38:512‐518. 22951175 10.1590/s1677-55382012000400011

[24] Keihani S , Wright LN , Alder NJ , et al. Baseline gonadotropin levels and testosterone response in hypogonadal men treated with clomiphene citrate. Urology. 2020;142:119‐124. 32353397 10.1016/j.urology.2020.04.074 PMC7749702

[25] Zitzmann M , Faber S , Nieschlag E . Association of specific symptoms and metabolic risks with serum testosterone in older men. J Clin Endocrinol Metabol. 2006;91(11):4335‐4343. 10.1210/jc.2006-0401 16926258

[26] Moskovic DJ , Katz DJ , Akhavan A , Park K , Mulhall JP . Clomiphene citrate is safe and effective for long‐term management of hypogonadism. BJU Int. 2012;110:1524‐1528. 22458540 10.1111/j.1464-410X.2012.10968.x

[27] Taylor F , Levine L . Clomiphene citrate and testosterone gel replacement therapy for male hypogonadism: efficacy and treatment costs. J Sex Med. 2010;7:269‐276. 19694928 10.1111/j.1743-6109.2009.01454.x

[28] Marconi M , Souper R , Hartmann J , Alvarez M , Fuentes I , Guarda FJ . Clomiphene citrate treatment for late onset hypogonadism: rise and fall. Int Braz J Urol. 2016;42:1190‐1194. 27622282 10.1590/S1677-5538.IBJU.2016.0112 PMC5117976

[29] Mazzola CR , Katz DJ , Loghmanieh N , Nelson CJ , Mulhall JP . Predicting biochemical response to clomiphene citrate in men with hypogonadism. J Sex Med. 2014;11(9):2302‐2307. 24902614 10.1111/jsm.12592

[30] Guay AT , Jacobson J , Perez JB , Hodge MB , Velasquez E . Clomiphene increases free testosterone levels in men with both secondary hypogonadism and erectile dysfunction: who does and does not benefit? Int J Impot Res. 2003;15:156‐165. 12904801 10.1038/sj.ijir.3900981

[31] Patel DP , Brant WO , Myers JB , et al. The safety and efficacy of clomiphene citrate in hypoandrogenic and subfertile men. Int J Impot Res. 2015;27:221‐224. 26289907 10.1038/ijir.2015.21

[32] Liel Y . Clomiphene citrate in the treatment of idiopathic or functional hypogonadotropic hypogonadism in men: a case series and review of the literature. Endocr Pract. 2017;23:279‐287. 27849376 10.4158/EP161543.OR

[33] Raheem OA , Chen TT , Akula KP , et al. Efficacy of non‐testosterone–based treatment in hypogonadal men: a review. Sex Med Rev. 2021;9:381‐392. 33933392 10.1016/j.sxmr.2020.08.003

[34] Chandrapal JC , Nielson S , Patel DP , et al. Characterising the safety of clomiphene citrate in male patients through prostate‐specific antigen, haematocrit, and testosterone levels. BJU Int. 2016;118:994‐1000. 27226135 10.1111/bju.13546

[35] Wheeler KM , Smith RP , Kumar RA , Setia S , Costabile RA , Kavoussi PK . A comparison of secondary polycythemia in hypogonadal men treated with clomiphene citrate versus testosterone replacement: a multi‐institutional study. J Urol. 2017;197:1127‐1131. 27984109 10.1016/j.juro.2016.10.068

[36] An J , Cheetham TC , Van Den Eeden S . Testosterone replacement therapy patterns for aging males in a managed care setting. Clin Med Res. 2013;11:141.

[37] Kemmeren JM , Algra A , Meijers JCM , Bouma BN , Grobbee DE . Effects of second and third generation oral contraceptives and their respective progestagens on the coagulation system in the absence or presence of the factor V Leiden mutation. Thromb Haemost. 2002;87(2):199‐205. 11859850

[38] Kavoussi PK , Machen GL , Wenzel JL , et al. Medical treatments for hypogonadism do not significantly increase the risk of deep vein thrombosis over general population risk. Urology. 2019;124:127‐130. 30447269 10.1016/j.urology.2018.11.009

[39] Ribeiro RS , Abucham J . Clomiphene fails to revert hypogonadism in most male patients with conventionally treated nonfunctioning pituitary adenomas. Arq Bras Endocrinol Metabol. 2011;55:266‐271. 21779629 10.1590/s0004-27302011000400005

[40] Pasqualotto FF , Fonseca GP , Pasqualotto EB . Azoospermia after treatment with clomiphene citrate in patients with oligospermia. Fertil Steril. 2008;55:266‐271. 10.1016/j.fertnstert.2008.03.036 18555230

