# Supplementary material for: Clomiphene citrate: A potential alternative for testosterone therapy in hypogonadal males
Source: Endocrinol Diabetes Metab. 2023 Mar 30;6(3):e416. doi: 10.1002/edm2.416 (PMC10164424; doi:10.1002/edm2.416)
Supplement: Supplementary file 1 — Appendix S1–S2. [file EDM2-6-e416-s001.docx]

## Supplementary material

|  | **Before CC treatment** | **During treatment** |
| --- | --- | --- |
| **BMI, kg/m^2^** | 27.9 ± 4.4  (n = 100) | 28.1 ± 3.5  (n = 12) |
| **HbA1c, mmol/mol** | 36.5 (33.2-45.3)  (n = 16) | 36.0 (35.0-39.3)  (n = 26) |
| **Total cholesterol, mmol/L** | 5.5 ± 1.1  (n = 43) | 4.8 ± 1.2  (n = 41)* |
| **LDL cholesterol, mmol/L** | 3.3 ± 1.1  (n = 42) | 2.7 ± 1.1  (n = 39)* |
| **HDL cholesterol, mmol/L** | 1.1 (0.4)  (n = 44) | 1.06 (0.9-1.3)  (n = 42)* |
| **Triglycerides, mmol/L** | 1.8 (1.3-3.2)  (n = 44) | 1.9 (1.2-2.5)  (n = 41) |
| **non-HDL cholesterol, mmol/L** | 4.3 ± 1.1  (n = 41) | 3.7 ± 1.6  (n = 42)* |

**Appendix 1.** **Metabolic and lipid parameters before and during CC treatment.** Data tabulated in numbers of patients (%), mean ± SD or median (IQR)

BMI = body mass index, HbA1c = hemoglobin A1c, LDL = low-density lipoprotein, HDL = high-density lipoprotein, n = number of patients, CC = clomiphene citrate, SD = standard deviation, IQR = interquartile range

* p ≤ 0.05.

|  | **Before CC treatment** | **First measurement of CC treatment** | **1 year with CC treatment** | **2 year with CC treatment** | **3 year with CC treatment** | **4 year with CC treatment** | **5 year with CC treatment** | **6 year with CC treatment** | **7 year with CC treatment** | **8 year with CC treatment** |
| --- | --- | --- | --- | --- | --- | --- | --- | --- | --- | --- |
| **Hematocrit, L/L** | 0.44 ± 0.04  (n = 67) | 0.44 ± 0.05  (n = 82) | 0.45 ± 0.04*  (n = 41) | 0.45 ± 0.04*  (n = 29) | 0.45 ± 0.04  (n = 14) | 0.47 ± 0.02*  (n = 12) | 0.47 ± 0.02  (n = 4) | 0.44 (0.44-0.46)  (n = 3) | 0.50  (n = 1) | 0.50  (n = 1) |
| **Hemoglobin, mmol/L** | 9.3 (8.8-9.7)  (n = 75) | 9.5 (8.8-9.9)  (n = 88) | 9.3 ± 0.9  (n = 43) | 9.5 ± 0.8  (n = 32) | 9.5 ± 0.8  (n = 17) | 9.6 ± 0.5  (n = 15) | 9.6 ± 0.1  (n = 4) | 9.2 ± 0.4  (n = 3) | 10.5  (n = 1) | 10.7  (n = 1) |
| **Thrombocytes, x 10^9/L** | 239 ± 60  (n = 66) | 229 ± 64  (n = 79) | 229 ± 63  (n = 40) | 225 ± 57  (n = 24) | 207 ± 68*  (n = 14) | 210 ± 70  (n = 12) | 160 ± 39  (n = 3) | 198 ± 61  (n = 3) | 222  (n = 1) | 200  (n = 1) |
| **ALAT, U/L** | 28 (19-38)  (n = 56) | 26 (19-34)  (n = 63) | 24 (19-35)  (n = 32) | 31 ± 11  (n = 16) | 23 (22-51)  (n = 12) | 32 ± 16  (n = 9) | 28 (23-33)  (n = 2) | 26 (22-29)  (n = 2) | n.a. | n.a. |
| **ASAT, U/L** | 26 (23-31)  (n = 51) | 28 (22-32)  (n = 62) | 28 ± 10  (n = 29) | 27 ± 5  (n = 14) | 27 ± 8  (n = 11) | 23 ± 9  (n = 8) | 29 (22-35)  (n = 2) | 33 (24-42)  (n = 2) | n.a. | n.a. |
| **AF, U/L** | 80 (68-94)  (n = 52) | 73 (59-82)*  (n = 58) | 70 (55-84)  (n = 27) | 67 (60-104)  (n = 13) | 68 (55-76)  (n = 12) | 67 (61-77)  (n = 9) | 88 (79-97)  (n = 2) | 115 (114-115)  (n = 2) | n.a. | n.a. |
| **gGT, U/L** | 36 (25-60)  (n = 54) | 37 (25-59)  (n = 62) | 36 (29-65)  (n = 31) | 50 (29-115)  (n = 15) | 41 (30-84)  (n = 12) | 62 ± 44  (n = 9) | 37 (34-40)  (n = 2) | 72 (59-85)  (n = 2) | 22  (n = 1) | n.a. |
| **PSA, µg/L** | 0.7 (0.5-1.3)  (n = 69) | 0.8 (0.5-1.3)  (n = 74) | 0.9 (0.6-2.3)  (n = 28) | 0.9 (0.6-2.1)  (n = 22) | 0.8 (0.5-1.6)  (n = 16) | 0.8 (0.4-2.2)  (n = 6) | 0.9 (0.6-2.5)  (n = 3) | 1.1 ± 0.6  (n = 4) | 0.5  (n = 1) | n.a. |

**Appendix 2. Serum parameters before and during CC treatment.** Data are tabulated as mean ± SD or median (IQR).

ALAT = alanine amino transferases, ASAT = aspartate transaminase, AF = alkalic phosphatases, gGT = gamma-glutamyl transferase, PSA = prostate specific antigen, CC = clomiphene citrate, SD = standard deviation, IQR = interquartile range, n = number of patients, n.a. = not available, n.s. = not significant
* p ≤0.05
